# (5*S*,6*R*)-5-Methyl-6-phenyl-4-propyl-1,3,4-oxadiazinane-2-thione

**DOI:** 10.1107/S1600536809019382

**Published:** 2009-05-29

**Authors:** Joshua L. Kocher, Kate L. Edler, Barbara A. Bohling, George P. Nora, Carrie Stafford, Shawn R. Hitchcock, Gregory M. Ferrence

**Affiliations:** a CB 4160, Department of Chemistry, Illinois State University, Normal, IL 61790, USA

## Abstract

The title mol­ecule, C_13_H_18_N_2_OS, is an oxadiazinanthione derived from (1*R*,2*S*)-norephedrine. There are two molecules in the asymmetric. Both adopt roughly half-chair conformations; however, the 5-position carbon orients out of opposite faces of the oxadiazinanthiones plane in the two molecules. In the crystal structure, they are oriented as a dimer linked by a pair of N—H⋯S hydrogen bonds. The absolute configuration has been established from anomalous dispersion and confirms the known stereochemistry based on the synthetic procedure.

## Related literature

For background, see: Hitchcock *et al.* (2002 ▶, 2008 ▶); Trepanier *et al.* (1968 ▶). For related compounds, see: Burgeson *et al.* (2004 ▶); Casper, Blackburn *et al.* (2002 ▶); Casper, Burgeson *et al.* (2002 ▶); Cremer & Pople (1975 ▶); Ferrence *et al.* (2003 ▶); Hitchcock *et al.* (2001 ▶, 2004 ▶); Rodrigues *et al.* (2005 ▶, 2006 ▶); Squire *et al.* (2005 ▶);  Szczepura *et al.* (2004 ▶). For structural analysis, see: Boeyens (1978 ▶); Bruno *et al.* (2004 ▶);  Cremer & Pople (1975 ▶); Spek (2009 ▶).
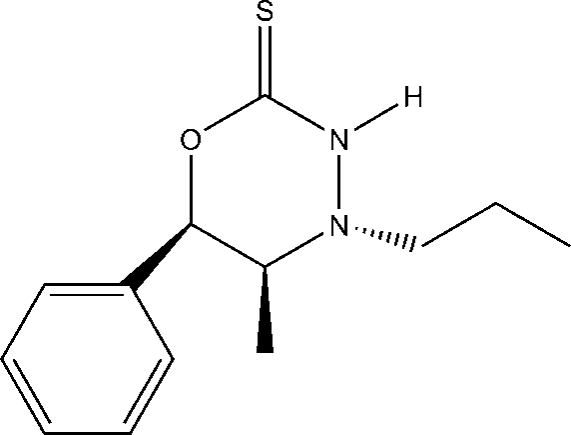


## Experimental

### 

#### Crystal data

C_13_H_18_N_2_OS
*M* *_r_* = 250.36
Monoclinic, 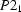

*a* = 12.5888 (6) Å
*b* = 8.0648 (4) Å
*c* = 14.2862 (7) Å
β = 112.4488 (7)°
*V* = 1340.51 (11) Å^3^
*Z* = 4
Mo *K*α radiation
μ = 0.23 mm^−1^
*T* = 193 K
0.45 × 0.3 × 0.26 mm

#### Data collection

Bruker SMART 1000 CCD diffractometer
Absorption correction: multi-scan (*SADABS* in *SAINT-Plus*; Bruker, 1999 ▶) *T*_min_ = 0.812, *T*_max_ = 0.943
10254 measured reflections
5344 independent reflections
5154 reflections with *I* > 2σ(*I*)
*R*_int_ = 0.016

#### Refinement

*R*[*F* ^2^ > 2σ(*F* ^2^)] = 0.028
*wR*(*F* ^2^) = 0.076
*S* = 1.03
5344 reflections
315 parameters
1 restraint
H atoms treated by a mixture of independent and constrained refinement
Δρ_max_ = 0.23 e Å^−3^
Δρ_min_ = −0.19 e Å^−3^
Absolute structure: Flack (1983 ▶)
Flack parameter: 0.03 (4)

### 

Data collection: *SMART* (Bruker, 1999 ▶); cell refinement: *SAINT* (Bruker, 1999 ▶); data reduction: *SAINT*; program(s) used to solve structure: *SIR2004* (Burla *et al.*, 2005 ▶); program(s) used to refine structure: *SHELXL97* (Sheldrick, 2008 ▶); molecular graphics: *ORTEP-3* for Windows (Farrugia, 1997 ▶) and *Mercury* (Macrae *et al.*, 2008 ▶); software used to prepare material for publication: *WinGX* (Farrugia, 1999 ▶) and *publCIF* (McMahon & Westrip, 2008 ▶).

## Supplementary Material

Crystal structure: contains datablocks global, I. DOI: 10.1107/S1600536809019382/fj2214sup1.cif

Structure factors: contains datablocks I. DOI: 10.1107/S1600536809019382/fj2214Isup2.hkl

Additional supplementary materials:  crystallographic information; 3D view; checkCIF report

**Fig. 3.**
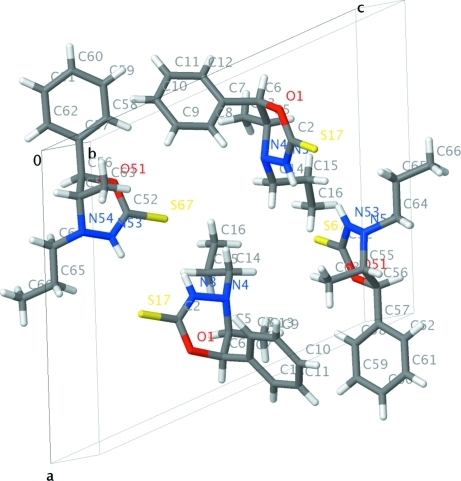
Jmol enhanced figure of (5S,6R)-5-Methyl-6-phenyl-4-propyl-1,3,4-oxadiazinane-2-thione.

Enhanced figure: interactive version of Fig. 3

## Figures and Tables

**Table 1:** Hydrogen-bond geometry (Å, °)

*D*—H⋯*A*	*D*—H	H⋯*A*	*D*⋯*A*	*D*—H⋯*A*
N3—H3⋯S67	0.86 (2)	2.48 (2)	3.3257 (14)	167 (2)
N53—H53⋯S17	0.82 (2)	2.56 (2)	3.3711 (15)	167 (2)

**Fig. 1.**
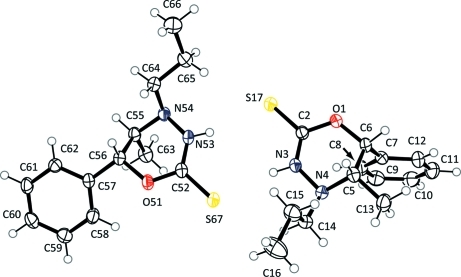
ORTEP view of C11H18N2OS showing the atomic numbering scheme and the two molecules in the asymmetric unit. Ellipsoids shown at 50% probability displacement.

**Fig. 2.**
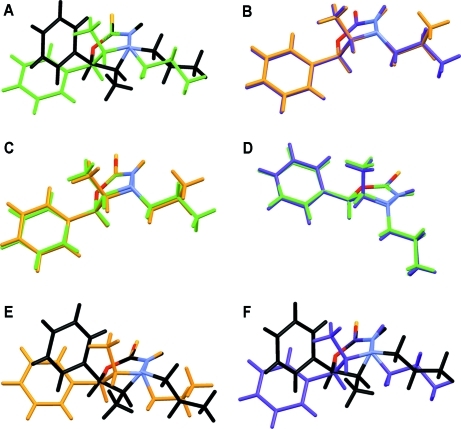
Mercury overlays of (A) molecules 1 and 2 of the title compound I; (B) molecules 1 and 2 of compound II (C11H18N2O2); (C—F) the four other possible permutations. Molecule 1 of the title compound is shown in green; molecule 2 of the title compound is shown in black; molecule 1 of compound II is shown in yellow; molecule 2 of compound II is shown in purple. For all overlays, hydrogen atoms are included.

## supplementary crystallographic information

### Comment

The heterocyclic structure of 1,3,4-oxadiazinan-2-one compounds have been known for nearly forty years, however the related compound 3,4,5,6-tetrahydro-2*H*-1,3,4-oxadiazinan-2-thione has just within the last 10 years been synthesized and studied (Trepanier *et al.*, 1968; Hitchcock *et al.*, 2002). Other oxadiazinanones, but no oxadiazinanthiones, have been reported and studied. The oxadiazinanone structures previously reported by Hitchcock and co-workers all contain a carbonyl group attached at the N3 position, which is believed to have an impact on the ring conformation (Burgeson *et al.*, 2004; Casper, Blackburn *et al.*, 2002; Casper, Burgeson *et al.*, 2002; Ferrence *et al.*, 2003; Hitchcock *et al.*, 2001, 2004, 2008; Squire *et al.*, 2005; Szczepura *et al.*, 2004).

The title compound, I, whose molecular structure is shown in Fig. 1, crystallizes with two independent molecules in the asymmetric unit with the two residues forming N–H···S intermolecularly H-bonded dimers with N3—H3···S67 = 2.48 (2) Å and N53—H53···S17 = 2.56 (2) Å. This H-bonding motif is similar to the N–H···O H-bonded dimers observed in the analogous oxadiazinaneone (Szczepura *et al.*, 2004). A *Mogul* (Bruno *et al.* 2004) geometry check showed all non-H bond angles and distances to be normal. X-ray crystal structural data obtained for the oxadiazinan-2-thione proved to be interesting in the context of comparing it to related oxadiazinanone structures (Casper, Burgeson *et al.*, 2002).

In fact, the two independent molecules in the asymmetric unit possess the two most common conformations for oxadiazine rings. Both molecules adopt roughly half-chair conformations with the most dramatic difference being whether the C5 (or C55) carbon resides above or below the respective mean O1—C2—N3—N4—C6 (or O51—C52—N53—N54—C56) plane. Ring puckering analysis using *PLATON* (Spek, 2009; Cremer & Pople, 1975; Boeyens, 1978) indicates θ = 129.9 (2)° and Φ = 55.1 (2)° for the O1—C2—N3—N4—C5—C6 ring. This is consistent with a formal conformational assignment close to an idealized envelope. Such a conformation possesses a pseudo-axial C5-methyl group, a typical pseudo-equatorial C6-phenyl ring, and a typical pseudo-axial N4-methyl group. Analysis of the O51—C52—N53—N54—C55—C56 ring indicates θ = 58.4 (2)° and Φ = 255.8 (2)°. This is consistent with a formal conformational assignment halfway between envelope (^5^E) and half-chair (^5^H_6_). In contrast, this conformation includes a pseudo-equatorial C55-methyl, the typical pseudo-axial C56-phenyl ring, and interestingly, a pseudo-equatorial N54-methyl group. This conformation represents a departure from the previously obtained X-ray crystal structures for the related ephedrine based oxadiazinanone wherein the N4-methyl group has always been observed in a pseudo-axial position (Szczepura *et al.*, 2004). The differences in conformation are more clearly illustrated in Fig. 2 which depicts a variety of overlays of the crystallographically independent molecules in the title compound and those found in the closely related oxadiazinanone, molecule II, 3,4,5,6-tetrahydro-2*H*-1,3,4-oxadiazinan-2-one. Fig. 2A shows the overlay of both independent molecules found in I and clearly shows their differing conformations. Fig. 2B shows the overlay of both independent molecules found in II and clearly shows the ring conformations to be essentially equivalent with the difference between the molecules being only the orientation of the terminal methyl group of the N4 propyl group. Figs. 2C and 2D show the nearly identical ring conformations of one of the independent molecules of I and either of the independent molecules of II. Figs. 2E and 2F clearly show the distinct conformation of the other independent molecule of I when compared with either independent molecule of II. All of these overlays were prepared in Mercury using a three point least-squares fit of the O(1), C(2), and N(3) atoms only. The existence of multiple conformers for oxadiazinanones has been observed before by Rodrigues and coworkers (Rodrigues *et al.*, 2005; 2006). The conformational flexibility of these systems is believed to be due to the nature of the substituents on the ring system. In the case of the oxadiazinan-2-thione, the source of the conformational flexibility is not clear and remains to be determined by further experimentation.

A Jmol enhanced figure (Fig. 3) accompanies this article. This enhanced figure is designed to illustrate the 2_1_ screw axis present in the monoclinic space group *P*21. Selecting appropriate radio buttons will highlight crystallographically identical molecules which are related by propagation along the 2_1_ screw axis parallel to the *b* axis.

### Experimental

The title compound was synthesized by Hitchcock *et al.* (Hitchcock *et al.*, 2002).

### Refinement

All non-H atoms were refined anisotropically without disorder. All H atoms were initially identified through difference Fourier syntheses then removed and included in the refinement in the riding-model approximation except the amine H atom which was freely refined (C–H = 0.95, 0.98, 0.99 and 1.00 Å for Ar–H, CH_3_, CH_2_, and CH; *U*_iso_(H) = 1.2Ueq(C) except for methyl groups, where *U*_iso_(H) = 1.5Ueq(C)).

### Crystal data

**Table:**

C_13_H_18_N_2_OS	*F*(000) = 536
*M**_r_* = 250.36	*D*_x_ = 1.24 Mg m^−^^3^
Monoclinic, *P*2_1_	Mo *K*α radiation, λ = 0.71073 Å
Hall symbol: P 2yb	Cell parameters from 4757 reflections
*a* = 12.5888 (6) Å	θ = 2.7–26.4°
*b* = 8.0648 (4) Å	µ = 0.23 mm^−^^1^
*c* = 14.2862 (7) Å	*T* = 193 K
β = 112.4488 (7)°	Prism, colourless
*V* = 1340.51 (11) Å^3^	0.45 × 0.3 × 0.26 mm
*Z* = 4	

### Data collection

**Table:**

Bruker SMART 1000 CCD diffractometer	5154 reflections with *I* > 2σ(*I*)
graphite	*R*_int_ = 0.016
ω scans	θ_max_ = 26.4°, θ_min_ = 1.5°
Absorption correction: multi-scan (*SADABS* in *SAINT-Plus*; Bruker, 1999)	*h* = −15→15
*T*_min_ = 0.812, *T*_max_ = 0.943	*k* = −9→10
10254 measured reflections	*l* = −17→17
5344 independent reflections	

### Refinement

**Table:**

Refinement on *F*^2^	H atoms treated by a mixture of independent and constrained refinement
Least-squares matrix: full	*w* = 1/[σ^2^(*F*_o_^2^) + (0.0464*P*)^2^ + 0.1813*P*] where *P* = (*F*_o_^2^ + 2*F*_c_^2^)/3
*R*[*F*^2^ > 2σ(*F*^2^)] = 0.028	(Δ/σ)_max_ = 0.001
*wR*(*F*^2^) = 0.076	Δρ_max_ = 0.23 e Å^−^^3^
*S* = 1.03	Δρ_min_ = −0.19 e Å^−^^3^
5344 reflections	Absolute structure: Flack (1983)
315 parameters	Flack parameter: 0.03 (4)
1 restraint	

### Special details

**Table:**

Geometry. All e.s.d.'s (except the e.s.d. in the dihedral angle between two l.s. planes) are estimated using the full covariance matrix. The cell e.s.d.'s are taken into account individually in the estimation of e.s.d.'s in distances, angles and torsion angles; correlations between e.s.d.'s in cell parameters are only used when they are defined by crystal symmetry. An approximate (isotropic) treatment of cell e.s.d.'s is used for estimating e.s.d.'s involving l.s. planes.

### Fractional atomic coordinates and isotropic or equivalent isotropic displacement parameters (Å^2^)

**Table:**

	*x*	*y*	*z*	*U*_iso_*/*U*_eq_	
H3	0.5801 (17)	0.487 (3)	0.3637 (14)	0.037 (5)*	
H53	0.4291 (18)	0.298 (3)	0.1944 (15)	0.044 (6)*	
O1	0.82647 (9)	0.40738 (15)	0.38779 (8)	0.0317 (2)	
C2	0.71123 (12)	0.3987 (2)	0.35160 (11)	0.0266 (3)	
N3	0.65394 (10)	0.49797 (16)	0.38728 (9)	0.0277 (3)	
N4	0.70414 (10)	0.60668 (16)	0.47180 (9)	0.0260 (3)	
C5	0.81231 (12)	0.67014 (19)	0.46870 (12)	0.0284 (3)	
H5	0.7956	0.7334	0.4044	0.034*	
C6	0.88793 (11)	0.5206 (2)	0.47089 (11)	0.0281 (3)	
H6	0.9556	0.5635	0.458	0.034*	
C7	0.93458 (12)	0.4261 (2)	0.56893 (12)	0.0292 (3)	
C8	0.86992 (13)	0.3065 (2)	0.59413 (12)	0.0337 (3)	
H8	0.7935	0.2837	0.5487	0.04*	
C9	0.91621 (16)	0.2203 (2)	0.68504 (13)	0.0402 (4)	
H9	0.8718	0.1386	0.7015	0.048*	
C10	1.02754 (15)	0.2541 (3)	0.75165 (13)	0.0427 (4)	
H10	1.0599	0.1942	0.8134	0.051*	
C11	1.09113 (15)	0.3742 (3)	0.72841 (13)	0.0432 (4)	
H11	1.1666	0.3988	0.7751	0.052*	
C12	1.04608 (13)	0.4594 (2)	0.63761 (12)	0.0365 (4)	
H12	1.0912	0.5409	0.6219	0.044*	
C13	0.87021 (14)	0.7838 (2)	0.55843 (13)	0.0384 (4)	
H13A	0.8199	0.8784	0.5548	0.058*	
H13B	0.9431	0.8239	0.557	0.058*	
H13C	0.8851	0.7227	0.6214	0.058*	
C14	0.61975 (13)	0.7372 (2)	0.46634 (11)	0.0301 (3)	
H14A	0.6508	0.8031	0.5295	0.036*	
H14B	0.5488	0.6824	0.465	0.036*	
C15	0.58656 (15)	0.8565 (2)	0.37746 (13)	0.0386 (4)	
H15A	0.5536	0.794	0.3131	0.046*	
H15B	0.6557	0.916	0.3782	0.046*	
C16	0.4989 (2)	0.9805 (3)	0.38432 (19)	0.0642 (6)	
H16A	0.4772	1.0565	0.3265	0.096*	
H16B	0.5325	1.0438	0.4474	0.096*	
H16C	0.4306	0.9212	0.3835	0.096*	
S17	0.64745 (3)	0.25776 (5)	0.25998 (3)	0.03521 (10)	
O51	0.18836 (8)	0.39587 (13)	0.16424 (8)	0.0282 (2)	
C52	0.30313 (12)	0.39369 (19)	0.20313 (11)	0.0261 (3)	
N53	0.35868 (11)	0.29154 (17)	0.16570 (9)	0.0277 (3)	
N54	0.30882 (10)	0.15932 (16)	0.09668 (9)	0.0273 (3)	
C55	0.19357 (12)	0.12386 (19)	0.09575 (12)	0.0270 (3)	
H55	0.1528	0.0502	0.0366	0.032*	
C56	0.12635 (11)	0.28685 (18)	0.07989 (11)	0.0252 (3)	
H56	0.1237	0.3395	0.0156	0.03*	
C57	0.00531 (11)	0.27209 (18)	0.07641 (10)	0.0252 (3)	
C58	−0.02242 (13)	0.3144 (2)	0.15848 (11)	0.0331 (3)	
H58	0.0358	0.3553	0.2186	0.04*	
C59	−0.13361 (14)	0.2977 (2)	0.15410 (13)	0.0371 (4)	
H59	−0.1513	0.3264	0.211	0.045*	
C60	−0.21914 (13)	0.2390 (2)	0.06632 (13)	0.0355 (3)	
H60	−0.2956	0.2271	0.063	0.043*	
C61	−0.19292 (13)	0.1979 (2)	−0.01602 (13)	0.0334 (3)	
H61	−0.2517	0.1584	−0.0762	0.04*	
C62	−0.08151 (13)	0.21385 (19)	−0.01173 (12)	0.0289 (3)	
H62	−0.0642	0.1851	−0.0688	0.035*	
C63	0.20153 (14)	0.0328 (2)	0.19114 (12)	0.0351 (3)	
H63A	0.2454	−0.0698	0.1975	0.053*	
H63B	0.124	0.0061	0.1873	0.053*	
H63C	0.2403	0.1034	0.2503	0.053*	
C64	0.31096 (13)	0.1969 (2)	−0.00361 (11)	0.0323 (3)	
H64A	0.2733	0.3053	−0.0271	0.039*	
H64B	0.2663	0.1114	−0.0525	0.039*	
C65	0.43186 (14)	0.2023 (3)	−0.00219 (13)	0.0449 (5)	
H65A	0.4725	0.0983	0.0277	0.054*	
H65B	0.4743	0.2957	0.0408	0.054*	
C66	0.43043 (18)	0.2236 (4)	−0.10884 (15)	0.0600 (6)	
H66A	0.5095	0.226	−0.1058	0.09*	
H66B	0.3918	0.3277	−0.1379	0.09*	
H66C	0.3891	0.1306	−0.1513	0.09*	
S67	0.36887 (3)	0.52486 (5)	0.29914 (3)	0.03515 (10)	

### Atomic displacement parameters (Å^2^)

**Table:**

	*U*^11^	*U*^22^	*U*^33^	*U*^12^	*U*^13^	*U*^23^
O1	0.0211 (5)	0.0432 (7)	0.0292 (5)	0.0010 (4)	0.0078 (4)	−0.0076 (5)
C2	0.0228 (7)	0.0307 (8)	0.0260 (7)	−0.0016 (6)	0.0090 (6)	0.0002 (6)
N3	0.0196 (6)	0.0315 (7)	0.0300 (6)	−0.0015 (5)	0.0072 (5)	−0.0047 (5)
N4	0.0235 (6)	0.0285 (7)	0.0252 (6)	−0.0016 (5)	0.0083 (5)	−0.0027 (5)
C5	0.0240 (7)	0.0301 (8)	0.0301 (7)	−0.0016 (6)	0.0093 (6)	0.0020 (6)
C6	0.0196 (6)	0.0349 (8)	0.0288 (7)	−0.0049 (6)	0.0081 (5)	−0.0034 (7)
C7	0.0230 (7)	0.0330 (8)	0.0310 (8)	0.0022 (6)	0.0096 (6)	−0.0041 (6)
C8	0.0276 (7)	0.0352 (8)	0.0355 (8)	−0.0006 (6)	0.0089 (6)	−0.0010 (7)
C9	0.0446 (10)	0.0385 (10)	0.0410 (9)	0.0034 (7)	0.0201 (8)	0.0034 (8)
C10	0.0461 (9)	0.0476 (10)	0.0314 (8)	0.0139 (9)	0.0112 (7)	0.0050 (8)
C11	0.0313 (8)	0.0539 (11)	0.0350 (9)	0.0074 (8)	0.0021 (7)	−0.0035 (8)
C12	0.0255 (8)	0.0433 (9)	0.0364 (8)	−0.0012 (7)	0.0070 (6)	−0.0025 (7)
C13	0.0339 (8)	0.0358 (9)	0.0408 (9)	−0.0067 (7)	0.0089 (7)	−0.0054 (7)
C14	0.0286 (7)	0.0344 (8)	0.0282 (7)	0.0022 (6)	0.0119 (6)	−0.0039 (6)
C15	0.0381 (9)	0.0367 (9)	0.0407 (9)	0.0073 (7)	0.0146 (7)	0.0047 (7)
C16	0.0586 (13)	0.0610 (15)	0.0805 (15)	0.0297 (11)	0.0349 (12)	0.0218 (12)
S17	0.02566 (18)	0.0418 (2)	0.0374 (2)	−0.00325 (16)	0.01108 (15)	−0.01413 (17)
O51	0.0194 (5)	0.0265 (5)	0.0336 (6)	−0.0015 (4)	0.0045 (4)	−0.0038 (4)
C52	0.0217 (7)	0.0280 (8)	0.0261 (7)	−0.0011 (6)	0.0063 (6)	0.0034 (6)
N53	0.0190 (6)	0.0345 (7)	0.0256 (6)	−0.0024 (5)	0.0040 (5)	−0.0028 (5)
N54	0.0221 (6)	0.0291 (7)	0.0279 (6)	−0.0005 (5)	0.0063 (5)	−0.0024 (5)
C55	0.0228 (7)	0.0250 (7)	0.0307 (8)	−0.0020 (6)	0.0073 (6)	−0.0007 (6)
C56	0.0222 (7)	0.0258 (7)	0.0250 (6)	−0.0022 (6)	0.0062 (5)	−0.0001 (6)
C57	0.0209 (6)	0.0241 (7)	0.0272 (7)	−0.0002 (5)	0.0053 (5)	0.0020 (6)
C58	0.0273 (7)	0.0420 (9)	0.0256 (7)	−0.0035 (6)	0.0052 (6)	0.0004 (6)
C59	0.0327 (8)	0.0471 (10)	0.0348 (8)	−0.0014 (7)	0.0164 (7)	−0.0006 (7)
C60	0.0234 (7)	0.0362 (9)	0.0473 (9)	−0.0019 (6)	0.0139 (7)	−0.0004 (8)
C61	0.0229 (7)	0.0315 (8)	0.0388 (8)	−0.0038 (6)	0.0039 (6)	−0.0063 (7)
C62	0.0262 (7)	0.0282 (8)	0.0303 (7)	−0.0003 (6)	0.0086 (6)	−0.0045 (6)
C63	0.0316 (7)	0.0327 (8)	0.0397 (8)	0.0012 (7)	0.0123 (6)	0.0072 (7)
C64	0.0262 (7)	0.0392 (9)	0.0285 (7)	−0.0016 (6)	0.0072 (6)	−0.0055 (7)
C65	0.0295 (8)	0.0701 (13)	0.0359 (9)	0.0009 (8)	0.0135 (7)	−0.0035 (9)
C66	0.0442 (10)	0.1014 (18)	0.0394 (10)	−0.0052 (11)	0.0218 (8)	−0.0079 (12)
S67	0.02293 (17)	0.0437 (2)	0.03399 (19)	−0.00201 (16)	0.00548 (14)	−0.01305 (17)

### Geometric parameters (Å, °)

**Table:**

O1—C2	1.3432 (17)	O51—C52	1.3355 (17)
O1—C6	1.4627 (18)	O51—C56	1.4548 (16)
C2—N3	1.305 (2)	C52—N53	1.319 (2)
C2—S17	1.6873 (15)	C52—S67	1.6788 (15)
N3—N4	1.4294 (17)	N53—N54	1.4250 (18)
N3—H3	0.86 (2)	N53—H53	0.82 (2)
N4—C5	1.4711 (18)	N54—C55	1.4739 (18)
N4—C14	1.476 (2)	N54—C64	1.475 (2)
C5—C13	1.517 (2)	C55—C63	1.517 (2)
C5—C6	1.530 (2)	C55—C56	1.533 (2)
C5—H5	1	C55—H55	1
C6—C7	1.503 (2)	C56—C57	1.5101 (18)
C6—H6	1	C56—H56	1
C7—C8	1.396 (2)	C57—C58	1.388 (2)
C7—C12	1.397 (2)	C57—C62	1.3963 (19)
C8—C9	1.390 (2)	C58—C59	1.384 (2)
C8—H8	0.95	C58—H58	0.95
C9—C10	1.388 (3)	C59—C60	1.388 (2)
C9—H9	0.95	C59—H59	0.95
C10—C11	1.375 (3)	C60—C61	1.378 (2)
C10—H10	0.95	C60—H60	0.95
C11—C12	1.384 (2)	C61—C62	1.386 (2)
C11—H11	0.95	C61—H61	0.95
C12—H12	0.95	C62—H62	0.95
C13—H13A	0.98	C63—H63A	0.98
C13—H13B	0.98	C63—H63B	0.98
C13—H13C	0.98	C63—H63C	0.98
C14—C15	1.519 (2)	C64—C65	1.515 (2)
C14—H14A	0.99	C64—H64A	0.99
C14—H14B	0.99	C64—H64B	0.99
C15—C16	1.520 (3)	C65—C66	1.527 (3)
C15—H15A	0.99	C65—H65A	0.99
C15—H15B	0.99	C65—H65B	0.99
C16—H16A	0.98	C66—H66A	0.98
C16—H16B	0.98	C66—H66B	0.98
C16—H16C	0.98	C66—H66C	0.98
			
C2—O1—C6	120.25 (12)	C52—O51—C56	119.32 (11)
N3—C2—O1	119.64 (13)	N53—C52—O51	119.77 (13)
N3—C2—S17	123.11 (11)	N53—C52—S67	123.55 (11)
O1—C2—S17	117.25 (11)	O51—C52—S67	116.68 (11)
C2—N3—N4	125.07 (12)	C52—N53—N54	125.90 (12)
C2—N3—H3	117.2 (13)	C52—N53—H53	113.1 (15)
N4—N3—H3	117.1 (13)	N54—N53—H53	120.0 (15)
N3—N4—C5	107.68 (11)	N53—N54—C55	108.87 (11)
N3—N4—C14	108.40 (11)	N53—N54—C64	110.61 (12)
C5—N4—C14	113.96 (12)	C55—N54—C64	114.47 (11)
N4—C5—C13	109.66 (13)	N54—C55—C63	111.02 (12)
N4—C5—C6	107.52 (12)	N54—C55—C56	108.73 (12)
C13—C5—C6	111.41 (12)	C63—C55—C56	113.03 (13)
N4—C5—H5	109.4	N54—C55—H55	108
C13—C5—H5	109.4	C63—C55—H55	108
C6—C5—H5	109.4	C56—C55—H55	108
O1—C6—C7	109.73 (13)	O51—C56—C57	106.89 (11)
O1—C6—C5	110.08 (11)	O51—C56—C55	107.73 (11)
C7—C6—C5	115.94 (12)	C57—C56—C55	115.50 (12)
O1—C6—H6	106.9	O51—C56—H56	108.9
C7—C6—H6	106.9	C57—C56—H56	108.9
C5—C6—H6	106.9	C55—C56—H56	108.9
C8—C7—C12	118.68 (15)	C58—C57—C62	118.80 (13)
C8—C7—C6	122.14 (13)	C58—C57—C56	121.94 (12)
C12—C7—C6	119.17 (14)	C62—C57—C56	119.26 (13)
C9—C8—C7	120.61 (15)	C59—C58—C57	120.97 (14)
C9—C8—H8	119.7	C59—C58—H58	119.5
C7—C8—H8	119.7	C57—C58—H58	119.5
C10—C9—C8	119.73 (17)	C58—C59—C60	119.77 (15)
C10—C9—H9	120.1	C58—C59—H59	120.1
C8—C9—H9	120.1	C60—C59—H59	120.1
C11—C10—C9	120.09 (16)	C61—C60—C59	119.83 (14)
C11—C10—H10	120	C61—C60—H60	120.1
C9—C10—H10	120	C59—C60—H60	120.1
C10—C11—C12	120.52 (16)	C60—C61—C62	120.54 (14)
C10—C11—H11	119.7	C60—C61—H61	119.7
C12—C11—H11	119.7	C62—C61—H61	119.7
C11—C12—C7	120.34 (17)	C61—C62—C57	120.09 (14)
C11—C12—H12	119.8	C61—C62—H62	120
C7—C12—H12	119.8	C57—C62—H62	120
C5—C13—H13A	109.5	C55—C63—H63A	109.5
C5—C13—H13B	109.5	C55—C63—H63B	109.5
H13A—C13—H13B	109.5	H63A—C63—H63B	109.5
C5—C13—H13C	109.5	C55—C63—H63C	109.5
H13A—C13—H13C	109.5	H63A—C63—H63C	109.5
H13B—C13—H13C	109.5	H63B—C63—H63C	109.5
N4—C14—C15	117.29 (12)	N54—C64—C65	112.58 (13)
N4—C14—H14A	108	N54—C64—H64A	109.1
C15—C14—H14A	108	C65—C64—H64A	109.1
N4—C14—H14B	108	N54—C64—H64B	109.1
C15—C14—H14B	108	C65—C64—H64B	109.1
H14A—C14—H14B	107.2	H64A—C64—H64B	107.8
C14—C15—C16	109.73 (16)	C64—C65—C66	111.17 (15)
C14—C15—H15A	109.7	C64—C65—H65A	109.4
C16—C15—H15A	109.7	C66—C65—H65A	109.4
C14—C15—H15B	109.7	C64—C65—H65B	109.4
C16—C15—H15B	109.7	C66—C65—H65B	109.4
H15A—C15—H15B	108.2	H65A—C65—H65B	108
C15—C16—H16A	109.5	C65—C66—H66A	109.5
C15—C16—H16B	109.5	C65—C66—H66B	109.5
H16A—C16—H16B	109.5	H66A—C66—H66B	109.5
C15—C16—H16C	109.5	C65—C66—H66C	109.5
H16A—C16—H16C	109.5	H66A—C66—H66C	109.5
H16B—C16—H16C	109.5	H66B—C66—H66C	109.5
			
C6—O1—C2—N3	3.6 (2)	C56—O51—C52—N53	−0.9 (2)
C6—O1—C2—S17	−176.82 (11)	C56—O51—C52—S67	179.09 (10)
O1—C2—N3—N4	−7.4 (2)	O51—C52—N53—N54	−11.2 (2)
S17—C2—N3—N4	173.03 (11)	S67—C52—N53—N54	168.81 (11)
C2—N3—N4—C5	36.25 (18)	C52—N53—N54—C55	−15.85 (19)
C2—N3—N4—C14	159.97 (14)	C52—N53—N54—C64	110.75 (16)
N3—N4—C5—C13	−178.87 (12)	N53—N54—C55—C63	−74.51 (15)
C14—N4—C5—C13	60.85 (16)	C64—N54—C55—C63	161.14 (14)
N3—N4—C5—C6	−57.59 (14)	N53—N54—C55—C56	50.43 (15)
C14—N4—C5—C6	−177.87 (11)	C64—N54—C55—C56	−73.92 (16)
C2—O1—C6—C7	100.10 (15)	C52—O51—C56—C57	160.86 (12)
C2—O1—C6—C5	−28.65 (18)	C52—O51—C56—C55	36.15 (16)
N4—C5—C6—O1	55.69 (15)	N54—C55—C56—O51	−60.99 (14)
C13—C5—C6—O1	175.87 (12)	C63—C55—C56—O51	62.77 (15)
N4—C5—C6—C7	−69.59 (16)	N54—C55—C56—C57	179.65 (11)
C13—C5—C6—C7	50.59 (17)	C63—C55—C56—C57	−56.60 (17)
O1—C6—C7—C8	−43.15 (19)	O51—C56—C57—C58	−17.70 (19)
C5—C6—C7—C8	82.32 (19)	C55—C56—C57—C58	102.13 (17)
O1—C6—C7—C12	137.21 (14)	O51—C56—C57—C62	162.47 (13)
C5—C6—C7—C12	−97.32 (17)	C55—C56—C57—C62	−77.70 (17)
C12—C7—C8—C9	−1.0 (2)	C62—C57—C58—C59	0.7 (2)
C6—C7—C8—C9	179.35 (15)	C56—C57—C58—C59	−179.12 (15)
C7—C8—C9—C10	0.3 (3)	C57—C58—C59—C60	−0.4 (3)
C8—C9—C10—C11	1.0 (3)	C58—C59—C60—C61	−0.2 (3)
C9—C10—C11—C12	−1.6 (3)	C59—C60—C61—C62	0.4 (3)
C10—C11—C12—C7	0.9 (3)	C60—C61—C62—C57	−0.1 (2)
C8—C7—C12—C11	0.4 (2)	C58—C57—C62—C61	−0.5 (2)
C6—C7—C12—C11	−179.97 (16)	C56—C57—C62—C61	179.38 (14)
N3—N4—C14—C15	−65.19 (17)	N53—N54—C64—C65	66.76 (17)
C5—N4—C14—C15	54.69 (18)	C55—N54—C64—C65	−169.82 (14)
N4—C14—C15—C16	179.66 (16)	N54—C64—C65—C66	174.12 (18)

### Hydrogen-bond geometry (Å, °)

**Table:**

*D*—H···*A*	*D*—H	H···*A*	*D*···*A*	*D*—H···*A*
N3—H3···S67	0.86 (2)	2.48 (2)	3.3257 (14)	167 (2)
N53—H53···S17	0.82 (2)	2.56 (2)	3.3711 (15)	167 (2)
